# Achieving High Piezoelectric Performance across a Wide Composition Range in Tetragonal (Bi,Na)TiO_3_–BaTiO_3_ Films for Micro-electromechanical Systems

**DOI:** 10.1021/acsami.3c13302

**Published:** 2023-12-28

**Authors:** Keisuke Ishihama, Takao Shimizu, Kazuki Okamoto, Akinori Tateyama, Wakiko Yamaoka, Risako Tsurumaru, Shintaro Yoshimura, Yusuke Sato, Hiroshi Funakubo

**Affiliations:** † School of Materials and Chemical Technology, Tokyo Institute of Technology, Yokohama 226-8502, Japan; ‡ Research Center for Functional Materials, National Institute for Materials Science, Tsukuba 305-0044, Japan; § Technical Center, TDK corporation, Ichikawa, Chiba 272-8558, Japan; ∥ Material Research Center for Element Strategy, Tokyo Institute of Technology, Yokohama 226-8502, Japan

**Keywords:** (Bi, Na)TiO_3_–BaTiO_3_, Pb-free, piezoelectric film, domain switching, out-of-morphotropic phase boundary composition, tetragonal structure

## Abstract

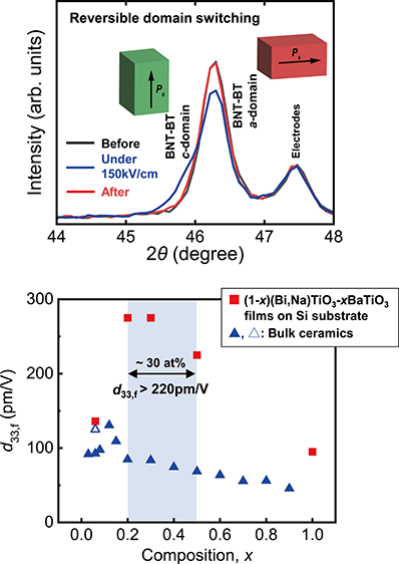

Tetragonal (1–*x*)(Bi,Na)TiO_3_–*x*BaTiO_3_ films exhibit enhanced piezoelectric properties due to domain switching over a wide composition range. These properties were observed over a significantly wider composition range than the morphotropic phase boundary (MPB), which typically has a limited composition range of 1–2%. The polarization axis was found to be along the in-plane direction for the tetragonal composition range *x* = 0.06–1.0, attributed to the tensile thermal strain from the substrate during cooling after the film formation. A “two-step increase” in remanent polarization against an applied maximum electric field was observed at the high-field region due to the domain switching, and a very high piezoelectric response (effective *d*_33_ value, denoted as *d*_33,f_) over 220 pm/V was achieved for a wide composition range of *x* = 0.2–0.5 with high tetragonality, exceeding previously reported values for bulk ceramics. Moreover, a transverse piezoelectric coefficient, *e*_31,f_, of 19 C/m^2^ measured using a cantilever structure was obtained for a composition range of at least 10 atom % (for both *x* = 0.2 and 0.3). This value is the highest reported for Pb-free piezoelectric thin films and is comparable to the best data for Pb-based thin films. Reversible domain switching eliminates the need for conventional MPB compositions, allowing an improvement in the piezoelectric properties over a wider composition range. This strategy could provide a guideline for the development of environmentally acceptable lead-free piezoelectric films with composition-insensitive piezoelectric performance to replace Pb-based materials with MPB composition, such as PZT.

## Introduction

Piezoelectric materials play a vital role in various applications including sensors, actuators, resonators, and vibration energy harvesters, particularly in the rapidly developing field of micro-electromechanical systems (MEMS).^1−4^ MEMS that use piezoelectric devices, also called piezoelectric MEMS, have advantages over their electrostatic and electromagnetic counterparts, such as a simple structure and small size.^5,6^ To date, Pb-based piezoelectric materials, such as Pb(Zr,Ti)O_3_ (PZT) and Pb(Mg_1/3_Nb_2/3_)O_3_–PbTiO_3_ (PMN–PT), have been preferred because of their excellent piezoelectric properties.^7,8^ However, environmental issues and stricter regulations such as the Restriction of Hazardous Substances (RoHS) directive have necessitated the development of alternative materials that do not contain toxic Pb.^9−11^ In the context of next-generation smart electronics, robotics, and the Internet of Things (IoT), advances in Pb-free piezoelectric thin films are essential for addressing the requirements of high-performance, energy-efficient devices while aligning with sustainable development practices.^12−15^

In the past, various strategies have been employed to improve the piezoelectric properties, including lattice contribution enhancement, domain reconstruction, morphotropic phase boundary (MPB) engineering, and defect control.^9,16−19^ Among these, MPB engineering has achieved significant breakthroughs and established itself as an important research strategy. In particular, PZT exhibits excellent piezoelectric properties near the MPB composition, where the PbTiO_3_-rich tetragonal and PbZrO_3_-rich rhombohedral exist in approximately equal proportions. Therefore, Pb-free materials with MPB compositions have been actively studied as alternatives to PZT. Typical Pb-free MPB materials, including (K,Na)NbO_3_- and (Bi,Na)TiO_3_-based solid solutions, have been successfully used in bulk-ceramic applications.^20−22^

Despite remarkable progress in Pb-free piezoelectric ceramics, the development of film-based piezoelectric materials for MEMS applications has challenges. A primary obstacle arises from substrate clamping, which substantially diminishes the electromechanical response, owing to the constraint of in-plane deformation of the film.^23−27^ In addition, stress from the substrate alters the phase boundaries of piezoelectric materials, diverging from their original composition in bulk ceramics.^26^ Therefore, an accurate composition adjustment is necessary to optimize performance. Piezoelectric properties typically exhibit significant sensitivity to composition near the MPB, rendering the control of these properties challenging in thin films.^7,28^ Consequently, exploration of alternative strategies without MPB is crucial for developing piezoelectric materials that can overcome the limitations imposed by substrate clamping and stress-induced phase-boundary shifts, thereby enhancing the potential of thin-film piezoelectric materials for MEMS applications.

Domain switching has recently emerged as a promising approach for enhancing the piezoelectric properties in Pb-free piezoelectric thin films for MEMS applications. This strategy focuses on microdomain structure formation, which is critical for a substantial piezoelectric response of ferroelectric thin films. The application of an electric field causes domain switching, and upon removing the electric field, the domain structure returns to its original state owing to the clamping effect of the substrate. This leads to macroscopic film deformation and large piezoelectric properties. A high piezoelectric coefficient of 310 pm/V has been reported for a tetragonal Pb(Zr,Ti)O_3_ film using this domain-switching concept.^29,30^ The factors controlling the piezoelectric response due to domain switching are the lattice anisotropy and changes in the volume fraction of the domain. For instance, the mobility of similar elastic domains in magnetostrictive materials such as Ni–Mn–Ga alloys has been reported to be dominated by the lattice anisotropy and volume fraction of the ferroelastic domains.^31,32^ These results suggest that the mobility of the domains can be controlled by using external electric fields to enhance their contribution to the piezoelectric response. That is, if a film with a large volume fraction of in-plane polarized domains has appropriate tetragonality, a *c*/*a* ratio (where *c* and a are the lattice parameters along the polar axis, or *c*-axis, and nonpolar axis, or *a*-axis, respectively) can be prepared, and large piezoelectric properties can be obtained by domain switching even for tetragonal films that have an out-of-MPB composition. Nakajima et al. reported that the large *c*/*a* ratio of PbTiO_3_ (*c*/*a* ≈ 1.06) was decreased by creating a solid solution with PbZrO_3_ in ferroelectric PZT thin films, and a large piezoelectric response was obtained at a tetragonal composition near Zr/(Zr+Ti) = 0.4 (*c*/*a* ≈ 1.02), with relatively low tetragonality than that of PbTiO_3_.^29^

We focused on a Pb-free material system, (1–*x*)(Bi,Na)TiO_3_–*x*BaTiO_3_ (BNT-BT), which is a solid solution of tetragonal BaTiO_3_ and rhombohedral (Bi,Na)TiO_3_. The materials in the ceramics exhibited tetragonal symmetry over a wide composition range of *x* = 0.06–1.0.^33,34^ Our group had previously reported that a tetragonal 0.7(Bi,Na)TiO_3_–0.3BaTiO_3_ (*x* = 0.3) film prepared on a Si substrate exhibited considerably large transverse piezoelectric coefficients (*e*_31,f_ = 19 C/m^2^) owing to domain switching.^35^ Rao et al. reported that the tetragonality hardly changed over a wide composition range of approximately 30 atom % for *x* = 0.2–0.5.^33^ These results suggest the possibility that tetragonal (Bi,Na)TiO_3_–BaTiO_3_ films exhibit a large piezoelectric response over a wide composition range via domain switching. Taking the concept of domain switching into account, the choice of a material system showing a stable *c*/*a* ratio, such as (Bi,Na)TiO_3_–BaTiO_3_, is optimal for achieving high piezoelectric properties over a wide composition range, which is in contrast to the continuous change of the *c*/*a* ratio with the Zr/(Zr+Ti) ratio in the case of PZT. However, there are few studies on the systematic composition dependence of the ferroelectric and piezoelectric properties of the tetragonal composition side of (1-*x*)(Bi,Na)TiO_3_–*x*BaTiO_3_ films, except for the high characteristics MPB neighborhood composition, which was observed at *x* = 0.04–0.07 composition.^36−38^

In this study, we investigated the composition dependence of the piezoelectric performance of (1–*x*)(Bi,Na)TiO_3_–*x*BaTiO_3_ (*x* = 0.06, 0.2, 0.3, 0.5, and 1.0) films on a Si substrate in the tetragonal phase to develop Pb-free piezoelectric thin films with a large piezoelectric response. As a result, we confirmed an out-of-plane piezoelectric response of *d*_33.f_ higher than 220 pm/V, which exceeded the reported value for bulk ceramics in the composition region of *x* = 0.2–0.5.^39^ Furthermore, *e*_31,f_ of 19 C/m^2^ was confirmed for samples with cantilever structures in the composition range of at least 10 atom % for *x* = 0.2 and 0.3. This value is the highest reported for Pb-free piezoelectric thin films and is comparable to the reported data for Pb-based materials.^15^ Furthermore, importantly, the films exhibited a large property over a composition range several times wider than that of MPB, which has a limited compositional range of 1–2%. This enables obtaining steady properties for the deposited films despite their composition fluctuation. This is an advantage over films using the MPB composition, which require precise composition control, owing to the high composition sensitivity of the piezoelectric property. The present innovative concept of reversible domain switching allows for a departure from the concept of using conventional MPB compositions and allows for improved piezoelectric properties over a wider composition range. These results will expand the scope of research for piezoelectric materials, which has focused mainly on Pb-based materials, especially near MPB composition, for the last 70 years.

## Results and Discussion

**Figure 1:**
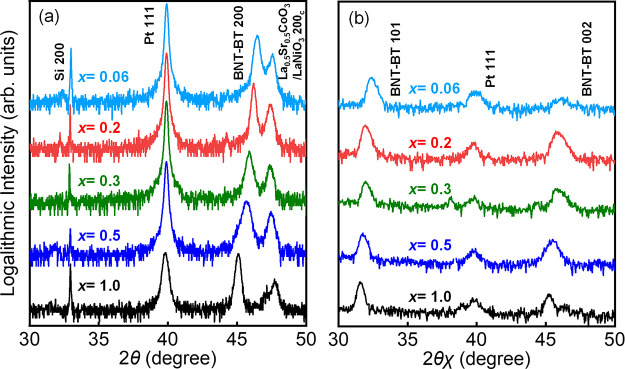
(a) Out-of-plane and (b) in-plane XRD profiles of prepared (Bi,Na)TiO_3_–BaTiO_3_ (*x* = 0.06–1.0) films on (100)_*c*_(La_0.5_Sr_0.5_)CoO_3_/(100)_*c*_ LaNiO_3_/ Pt/Ti/SiO_*x*_/(100)Si substrates.

Figure 1(a,b) shows the out-of-plane and in-plane X-ray diffraction (XRD) patterns of the 2.0 μm thick (1–*x*)(Bi,Na)TiO_3_–*x*BaTiO_3_ films with *x* = 0.06–1.0 on the Si substrate with buffer layers, respectively. Wider 2θ and 2θ*χ* range scan data are presented in Figure S1(a,b). The out-of-plane XRD patterns shown in Figures 1(a) and S1(a) in the Supporting Information and *h*00 or 00*l* diffraction peaks from tetragonal (Bi,Na)TiO_3_–BaTiO_3_ were observed together with *h*00_c_ diffraction peaks from other underlying perovskite layers with pseudocubic cells, such as LaNiO_3_ and (La_0.5_Sr_0.5_)CoO_3_. In addition, the shift of the (200) diffraction peaks to lower angles with an increasing *x* value without obvious different phase indicates the increase in the out-of-plane lattice parameter, *a*-axis, and the formation of solid solution as the bulk references (33,40). The in-plane GIXRD patterns shown in Figures 1(b) and S1(b) in the Supporting Information show the peaks derived from the perovskite structure. In addition, the presence of both {101} and {100} peaks in these in-plane measurements indicates that the in-plane direction is polycrystalline, thereby indicating a uniaxially oriented film.

Figure S2(a,b) shows surface and cross-sectional SEM images, respectively.

As shown in Figure S2(a), the thin film is composed of grains with almost uniform size. The random shape of the grains corresponds to the in-plane polycrystalline nature of the film. In addition, as shown in Figure S2(b), the film with a dense and no clear columnar structure was detected.

**Figure 2:**
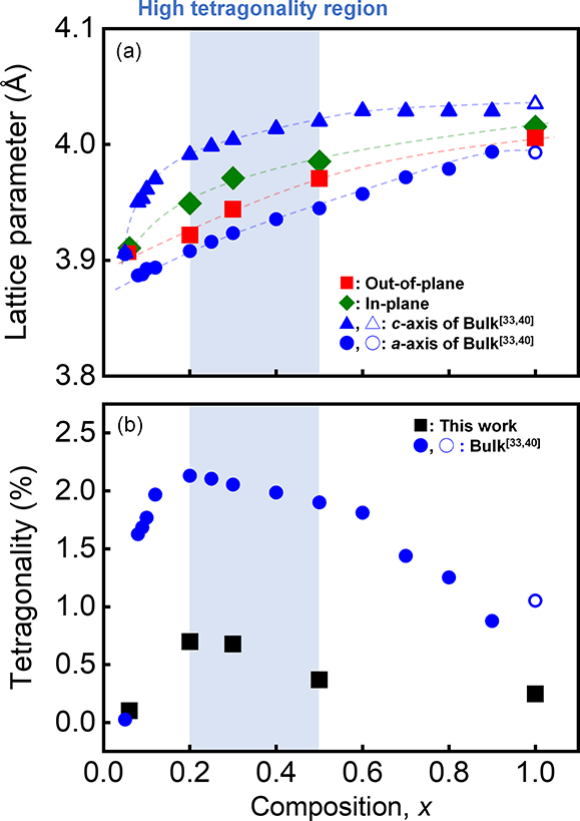
Composition (*x*) dependencies of (a) out-of-plane (squares) and in-plane (diamonds) lattice parameters and (b) the *c*/*a* ratio (squares) for (1–*x*)(Bi,Na)TiO_3_–*x*BaTiO_3_ films with *x* = 0.06–1.0. The closed^33^ and open^40^ circles and triangles represent the reported values for the powders.

Figure 2(a,b) illustrates the composition dependence of the out-of-plane and in-plane lattice parameters obtained from out-of-plane XRD θ–2θ and in-plane GIXRD scans along with the tetragonality, defined as {(out-of-plane lattice parameter)/(in-plane lattice parameter) – 1} and presented in Figure 2(b). The squares and diamonds represent the out-of-plane and in-plane lattice parameters, respectively, calculated from the {200} peak position of the out-of-plane XRD θ–2θ scan and the {002} peak positions on the in-plane GIXRD pattern, respectively. The previously reported *c*- and *a*-axes data for the sintered body, depicted using closed^33^ and open^40^ circles, and *c*/*a* ratios, depicted using triangles, are plotted in Figure 2(a,b), respectively.

The results shown in Figure 2(a) reveal that all of the films prepared in this study have smaller *c*-axis values and larger *a*-axis values than the bulk lattice parameters. It is already ascertained that this orientation did not dramatically change by changing the underlying (La,Sr)CoO_3_ to other bottom electrodes, such as SrRuO_3_ (not shown here).

As shown in Figure 2(b), the observed tetragonality was considerably smaller than the reported values for ceramics. Relatively higher tetragonality was observed in the approximate composition range of *x* = 0.2–0.5 among the films prepared in this study, indicated using a hatch in Figure 2, while the tetragonality of the film at *x* = 0.06 was nearly 0%. Considering the pseudocubic structure reported for (Bi,Na)TiO_3_–BaTiO_3_ at *x* = 0.04–0.07 in bulk ceramics, the results presented herein for the films were almost consistent with the reported results, and thus, the (Bi,Na)TiO_3_–BaTiO_3_ films reported herein have a tetragonal structure when *x* = 0.2–1.0.

According to our previous reports, tetragonal (Bi,Na)TiO_3_–BaTiO_3_ films (*x* = 0.06–1.0) deposited on SrTiO_3_ substrates are epitaxial films and were subjected to detailed XRD analysis.^41,42^ These results show that the volume fraction of the (100) orientation and non-180° domain fraction of the (100)/(001)-oriented ferroelectric films are determined by the thermal strain from the substrate; the thermal expansion coefficient of the SrTiO_3_ substrate is 10.9 × 10^–6^/K,^43^ which is larger than that of (Bi,Na)TiO_3_–BaTiO_3_ (approximately 6 × 10^–6^ /K).^33^ Thus, the (Bi,Na)TiO_3_–BaTiO_3_ film on the SrTiO_3_ substrate was confirmed to have a pure (001) orientation and *c*-domain structure with a polarization axis along the out-of-plane direction, which can be attributed to the in-plane compressive strain experienced during the cooling process after deposition. Conversely, Shimizu et al. reported that (Bi,Na)TiO_3_–BaTiO_3_ films deposited on Si substrates have a (100) orientation and *a*-domain structure with a polarization axis along the in-plane direction due to the in-plane tensile strain because the Si substrate has a thermal expansion coefficient smaller than that of the films,^35^ that is, 3.6 × 10^–6^/K.^44^ The in-plane lattice parameters were larger than the out-of-plane parameters for all films prepared in this study. These results suggest that the (Bi,Na)TiO_3_–BaTiO_3_ films on the Si substrate in this study are considered to be *a*-domain oriented films for all tetragonal compositions.

**Figure 3:**
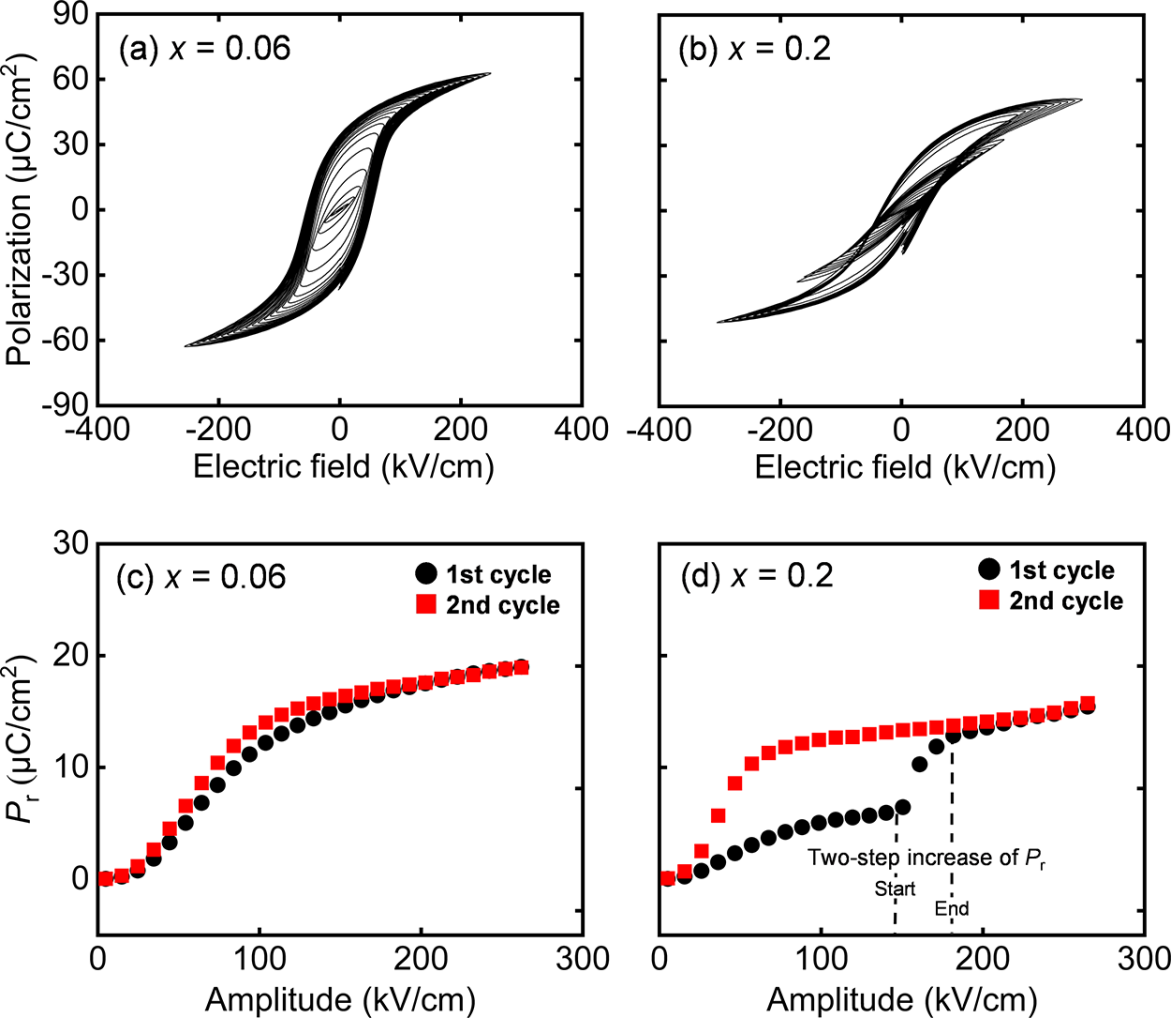
(a, b) *P*–*E* hysteresis curves for various amplitudes of the maximum electric field from the first cycle sweep-up and (c, d) the *P*_r_ value as a function of the amplitude of the maximum electric field for (1-*x*)(Bi,Na)TiO_3_–*x*BaTiO_3_ films with (c) *x* = 0.06 and (d) *x* = 0.2. The data from the first and second cycles of sweep-up are shown in panels (c) and (d) as closed circles and squares, respectively.

Figure 3 shows the measured electrical properties. Figure 3(a,b) illustrates the polarization–electric (*P*–*E*) curves measured at 10 kHz with various amplitudes of the triangular wave for (1–*x*)(Bi,Na)TiO_3_–*x*BaTiO_3_ films at (a) *x* = 0.06 and (b) *x* = 0.2, respectively, where the amplitudes increased sequentially. The measurement was conducted on a pristine electrode without any applied electric field. For these two compositions, the remanent polarization (*P*_r_) was well saturated above a high electric field amplitude of 200 kV/cm; however, the manner of saturation was different, particularly at a low electric field amplitude of <150 kV/cm. Small loops were observed at small amplitudes compared with those at large amplitudes for films with *x* = 0.2. Figure 3(c,d) shows the *P*_r_ value as a function of the amplitude for films with *x* = 0.06 and 0.2, respectively. These measurements were performed twice, from low to high electric fields, for each composition film, and the results for the first and second sweeps are indicated using black squares and red circles, respectively. The black-circle plots in Figure 3(c) indicate that the *P*_r_ value shows similar behavior in the first and second cycles for the film with *x* = 0.06, an increase with increasing electric field amplitude, and saturation above the coercive electric field, in agreement with the gradual change in the *P*–*E* loop in Figure 3(a). This behavior is typical of ferroelectric materials. Conversely, as represented by the circles in Figure 3(d), the *P*_r_ value of the film with *x* = 0.2 tends to saturate against the amplitude twice; the first saturation occurs at a relatively low value below approximately 150 kV/cm, and the second saturation occurs above 200 kV/cm through a rapid increase at 160–200 kV/cm.^30^ The value after the second saturation is 1.5–2 times larger than that after the first saturation.

No abrupt increase in the *P*_r_ value was observed during the second cycle. Consequently, the *P*_r_ value observed in the second cycle was larger than that in the first cycle, as shown in Figure 3(d), at low-field amplitudes such as 120 kV/cm. The measured amplitude dependencies of the *P*_r_ for all (1–*x*)(Bi,Na)TiO_3_–*x*BaTiO_3_ (*x* = 0.06–1.0) films in the first and second cycles are also plotted in Figure S2(a,b). Two-step increments in *P*_r_ are observed for films with high tetragonality in Figure 2(b), that is, those with *x* = 0.2, 0.3, and 0.5.

**Figure 4:**
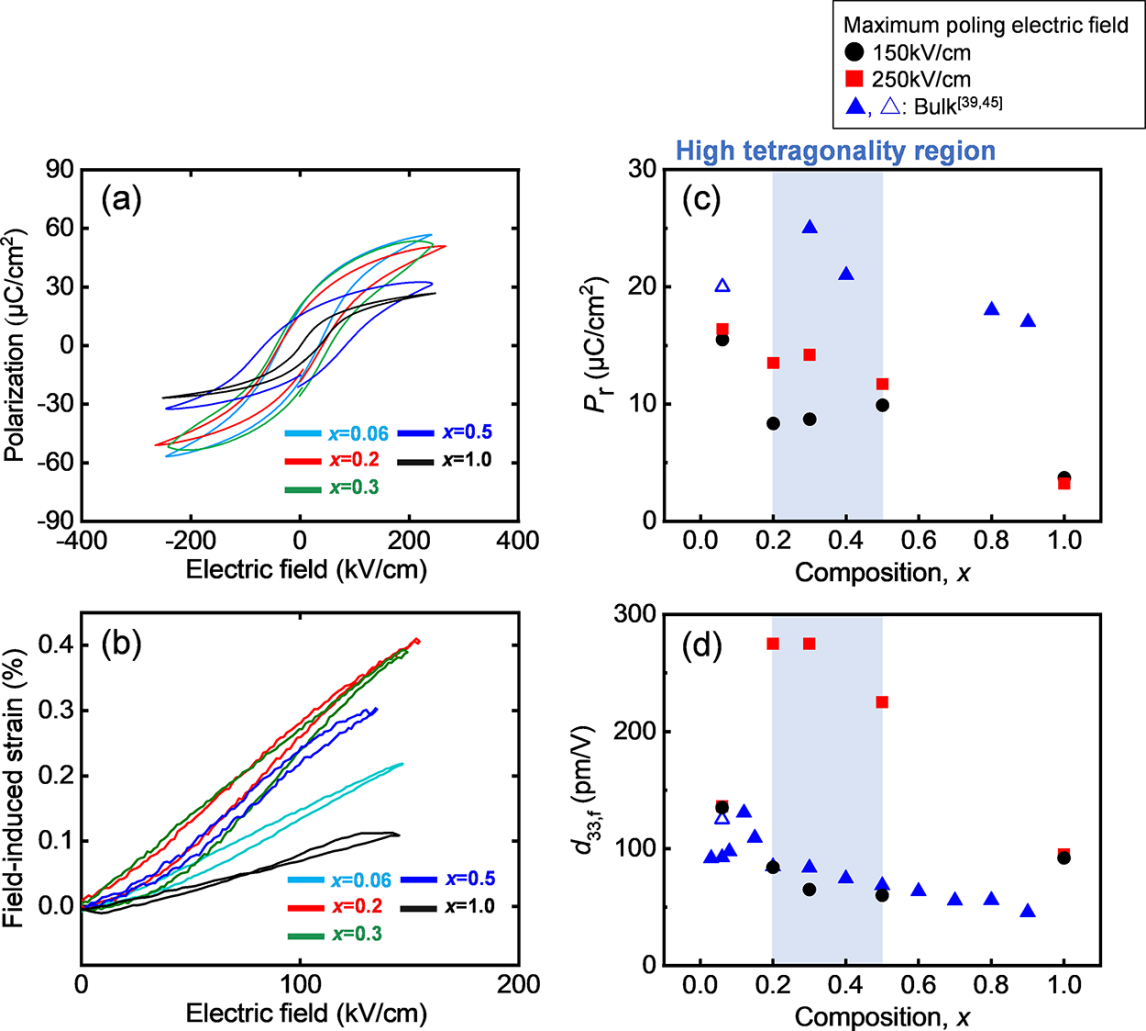
(a) Second-time sweep-up *P*–*E* curves measured at 10 kHz and the electric field amplitude of 250 kV/cm and (b) unipolar-driven strain–electric field (*S*–*E*) curves measured at 10 kHz and the amplitude of 150 kV/cm after the poling treatment by an amplitude of +250 kV/cm for (1–*x*)(Bi,Na)TiO_3_–*x*BaTiO_3_ (*x* = 0.06–1.0) films. Composition (*x*) dependencies of (c) *P*_r_ and (d) piezoelectric properties, *d*_33.f_, for an amplitude of 150 kV/cm after applying a poling electric field of 150 kV/cm (closed circles) and 250 kV/cm (closed squares). The closed^39^ and open^45^ circles and triangles are the reported values for the powders.

Figure 4(a) illustrates the *P*–*E* relationships measured at 10 kHz and the electric field amplitude of 250 kV/cm from the second cycle of sweep-up for (1–*x*)(Bi,Na)TiO_3_–*x*BaTiO_3_ (*x* = 0.06–1.0) films. Clear hysteresis loops originating from ferroelectricity were obtained for all films. In addition, Figure 4(b) shows the unipolar strain–electrical field (*S*–*E*) curves measured at 10 kHz and an amplitude of +150 kV/cm after the poling treatment with an amplitude of +250 kV/cm.

The composition dependence of *P*_r_ and piezoelectric properties, that is, the longitudinal effective piezoelectric response *d*_33.f_, at an applied electric field amplitude of 150 kV/cm is presented in Figure 4(c,d), respectively. In this study, *d*_33.f_ was defined as *S*_max_/*E*_max_, where *S*_max_ and *E*_max_ are the maximum strain and electric field, respectively. In Figure 4(c,d), the circles represent *P*_r_ and *d*_33.f_, at a 150 kV/cm electric field amplitude after poling at +150 kV/cm, while the squares represent data after poling at +250 kV/cm, that is, the circles and squares in Figure 4(c) correspond to the *P*_r_ values of the first and second cycles at a 150 kV/cm amplitude, respectively, presented in Figure S3.

In Figure 4(c), the composition dependence of the *P*_r_ value shows a continuous decrease as the *x* value deviates from the MPB composition, *x* ≈ 0.06, for both the first and second cycles. The *P*_r_ of the films was smaller than those reported for ceramics and *c*-axis oriented films on SrTiO_3_ substrates.^39,41^ This is mainly due to the *a*-axis orientation of these films and suppressed tetragonality in comparison with bulk ceramics, as shown in Figure 2(b).^39^ Furthermore, comparing the data for the first and second cycles of sweep-up in Figure 4(c) revealed that the films with *x* = 0.2, 0.3, and 0.5 exhibit a “two-step increase” in *P*_r_ value, as confirmed from the data presented in Figures 3 and S2. These films exhibit relatively high tetragonality values, as shown in Figure 2(b). In bulk ceramics, it has been reported that tetragonal and rhombohedral phases coexist at *x* = 0.05–0.07.^28^ In the present study, the remanent polarization value of the film with *x* = 0.06 is larger than that of the tetragonal films with *x* = 0.2–1.0 and the *a*-domain as a majority orientation, suggesting that the film with *x* = 0.06 is possible to include a rhombohedral phase that has a [111] polar axis. The observed tetragonality (axial ratio) of almost unity also supports the existence of a rhombohedral phase in the film with *x* = 0.06. In Figure 4(d), the composition dependence of *d*_33,f_ in the first cycle exhibits a trend similar to that of the ceramics, where the values decreased with the *x* value departing from the MPB composition, *x* ≈ 0.06. Conversely, in the second cycle, films with compositions of *x* = 0.2–0.5, where a two-step increase in *P*_r_ was observed in Figure S2(b), showed high *d*_33,f_ values beyond 220 pm/V. These values surpass the results for the film at *x* = 0.06, the composition closest to the MPB, and those previously reported for ceramics at *x* = 0.06 (*d*_33,f_ ≈ 140 pm/V) near MPB composition.^45^ In short, large *d*_33.f_ above 220 pm/V was obtained over a wide composition range of 30 atom % for tetragonal BNT-BT films far from the MPB composition and it exceeds the previously reported value for bulk ceramics with MPB composition, as expected. Here, Figure S4(a–e) shows the atomic force microscopy (AFM) images of the films with *x* = 0.06–1.0. The randomly arranged and uniformly shaped grains were observed for all films. These results may correspond to the in-plane polycrystalline characteristics of the films. The average roughness (*R*_a_) plotted against composition is shown in Figure S5. As shown in Figures 4(d) and S5, these results show no strong correlation between the composition dependences of *R*_a_ and *d*_33,f_.

Owing to the large thicknesses of the films, the misfit strains are considered to be almost relaxed during film deposition, which is introduced by the difference in the lattice parameters between the film and the substrate.^46,47^ Shimizu et al. explained that the release of tensile strain accumulated during cooling after deposition at the Curie temperature resulted in the formation of BNT-BT films with *a*-domain dominant structures having both *a*- and *c*-axes along the in-plane direction deposited on a Si substrate with a low thermal expansion coefficient.^29,35^ In contrast, compressive strain is generated upon cooling below *T*_C_ after film deposition, mainly owing to expansion in the *c*-axis domain structure along the in-plane direction. This strain is relieved by an increase in the domain wall owing to an external force, such as the application of an electric field, and *P*_r_, namely, the out-of-plane polarization component, increases as shown in Figure 3(d). This “two-step increase” of the *P*_r_ value appears to begin by applying an electric field of about 150 kV/cm and saturate at about 180 kV/cm. These results suggest that the domain wall remained after the removal of the electric field, and applying a small electric field is sufficient to activate wall motion. Therefore, the domain could be reversibly moved under an applied electric field, thereby enhancing the piezoelectric response.

**Figure 5:**
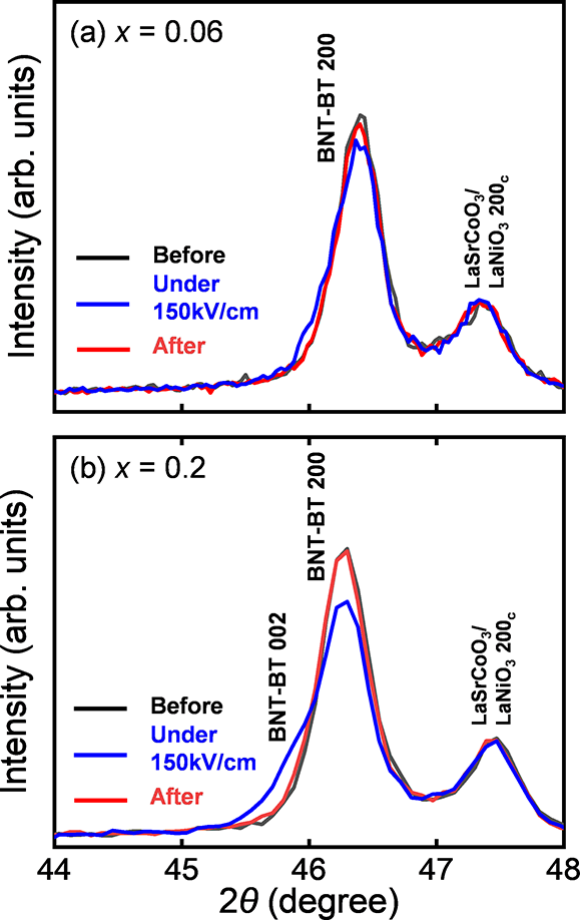
XRD θ–2θ patterns before (after poling) (red line), under (blue line), and after (green line) the application of an electric field of approximately 200 kV/cm for (1–*x*)(Bi,Na)TiO_3_–*x*BaTiO_3_ films with (a) *x* = 0.06 and (b) *x* = 0.2.

The *in situ* XRD was performed under an applied electric field to ascertain the mechanism of the large piezoresponse. Figure 5 compares the XRD patterns of the (1–*x*)(Bi,Na)TiO_3_–*x*BaTiO_3_ films before (black lines), under (blue lines), and after (red lines) the application of a + 150 kV/cm electric field for films with (a) *x* = 0.06 and (b) *x* = 0.2. As shown in Figure 5(a,b), the XRD patterns before and after the application of the electric field are almost the same for both films with *x* = 0.06 and 0.2, and thus, the crystal structure of the films are the same, with the in-plane polarized *a*-domain as the main orientation. In Figure 5(a), only the {200} peak appears in the diffraction pattern, even under the application of an electric field, and it hardly changes compared with the diffraction peaks before and after applying the electric field for the film with a *x* = 0.06 film. However, additional peaks located at an angle lower than the original position were observed under an electric field for the film with *x* = 0.2, as shown in Figure 5(b). Considering that the original peak position was identified as a {200} peak originating from the in-plane polarized domain, the novel peak can be identified as the {002} peak from the out-of-plane polarized domain. This suggests a change in the polarization direction when an electric field is applied; that is, the *a*-domain switches to the *c*-domain by applying an electric field. When the electric field was turned off, the XRD pattern was almost the same as that before the application of the electric field, suggesting that reversible domain switching occurred due to the application of the electric field. The piezoelectric response *d*_33.f_ from the domain switching can be estimated by using the peak-fitting method. The estimated volume fraction of the *c*-domain was approximately 33% under the electric field, as shown in Figure S6 and Table S1. On the basis of this change in the volume fraction, *d*_33,f_ ≈ 152 pm/V was calculated using the following equation^35^
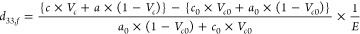
1where *E*, *V*_c_, *c*, and *a* represent the electric field, *c*-domain volume fraction, *c*-axis lattice parameter, and *a*-axis lattice parameter, respectively. The subscript “0” denotes no application of an electric field. The angle of incidence of the X-rays was not 90°, causing the beam to spread over the electrode of interest in an elliptical shape with a long diameter of 400 μm and a short diameter of 100 μm. Therefore, even at the best beam position, the XRD pattern comprised approximately 42% diffraction from outside the electrode, resulting in an underestimation of the *d*_33,f_ value. If the electrode diameter completely covered the beam diameter, *d*_33,f_ was 260 pm/V, which almost agreed with the results shown in Figure 4(b,d). This result suggests that domain switching is the origin of the large piezoelectric response of the films with *x* = 0.2, as shown in Figure 4, similar to that of films with *x* = 0.3, as demonstrated in our previous study.^35^ This can be explained by the similar tetragonality of the two films, as shown in Figure 2(b).

Finally, the transverse piezoelectric coefficients, *e*_31,f_, which are widely used to characterize the piezoelectric properties of thin films, were measured to compare the piezoelectric properties of films prepared on other substrates and films of other materials with *x* = 0.2. The *e*_31,f_ were calculated from the curvature of the cantilever, owing to the actuation of the piezoelectric film on the beam.^48,49^

**Figure 6:**
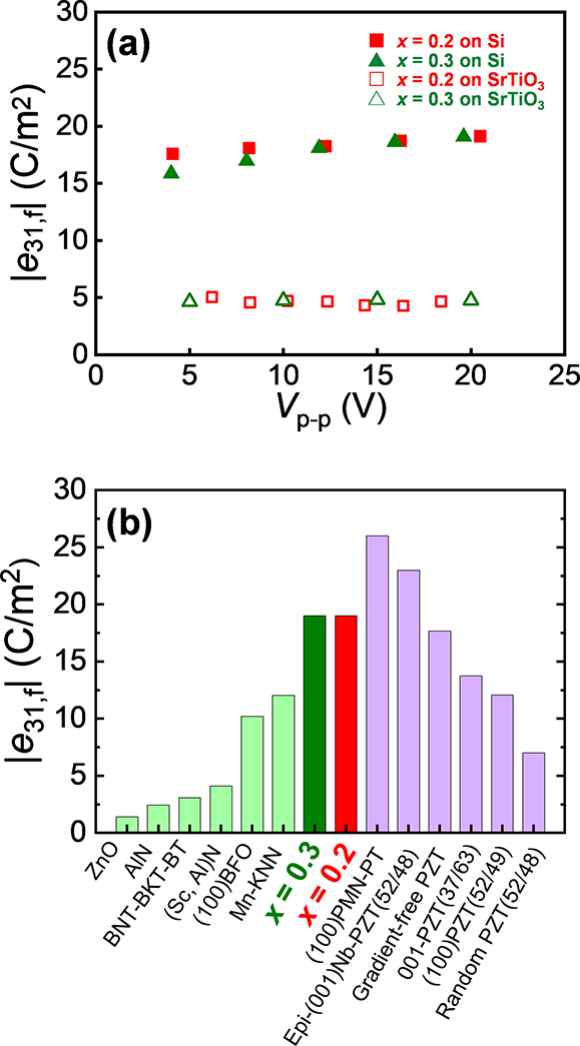
(a) *e*_31,f_ values as a function of the measurement voltage (peak to peak) for (Bi,Na)TiO_3_–BaTiO_3_ films, with *x* = 0.2 and 0.3, deposited on the Si substrates. (b) *e*_31, f_ values obtained in this study along with the reported data for other piezoelectric films.^15^

Figure 6(a) displays the estimated *e*_31,f_ versus the measured voltage, with closed and open squares representing the results for the Si and SrTiO_3_ substrates for films with *x* = 0.2, respectively, while closed and open triangles represent the results for the Si and SrTiO_3_ substrates for films with *x* = 0.3. The films on SrTiO_3_ show *e*_31,f_ values of 4–5 C/m^2^ for both compositions, which are comparable to those of other perovskite epitaxial films.^15^ In contrast, the films on the Si substrate exhibited a high *e*_31,f_ value of 19 C/m^2^ for both film compositions.^35,50^ No clear “two-step increase” was detected in the dependence of *e*_31,f_ on an applied electric field. This may be due to the piezoelectric signal at the first step being too small and/or the differences in the measurement frequency and electrode size for the *d*_33,f_ and *e*_31,f_ measurements. However, as shown in Figures 4 and 6, both *d*_33,f_ and *e*_31,f_ show large values, suggesting that domain switching was already completed when the 20 V pulsed wave was applied.

Figure 6(b) presents a comparison of the *e*_31,f_ values obtained in this study with those of previous studies on Pb-based perovskite films and Pb-free materials.^15^ The *e*_31,f_ values of the (1–*x*)(Bi,Na)TiO_3_–*x*BaTiO_3_ (*x* = 0.2 and 0.3^35^) films are the highest among the Pb-free materials, surpassing those of most Pb-based films, except for the relaxor Pb(Mg,Nb)O_3_–PbTiO_3_ and Nb-doped Pb(Zr,Ti)O_3_ films. More importantly, an *e*_31,f_ value of 19 C/m^2^ was obtained for a composition range of at least 10 atom % range (for both of *x* = 0.2 and 0.3), which is much wider than a morphotropic phase boundary with a limited composition range of 1–2%.

These findings imply that an improved piezoelectric response using domain switching can pave the way for practical applications in various devices, including those based on MEMS technology, owing to the large piezoresponse over a wide composition range.

## Conclusions

Tetragonal (1–*x*)(Bi,Na)TiO_3_–*x*BaTiO_3_ films were deposited on Si substrates over a wide composition range (*x* = 0.06, 0.2, 0.3, 0.5, and 1.0), and the polarization axis was principally aligned in the in-plane direction owing to the tensile thermal strain from the substrate. XRD measurements revealed a trend of composition dependency for the tetragonality, similar to that for bulk ceramics, with a maximum value at approximately *x* = 0.2–0.5; however, its absolute value was smaller than that reported for bulk ceramics. For the high tetragonality composition region, we observed a “two-step increase” in remanent polarization due to domain rearrangement under a high-field amplitude and an exceptional piezoelectric response (*d*_33,f_ > ∼200 pm/V), surpassing reported values of 30% for bulk ceramics in the composition range 0.2–0.5. *In situ* XRD analysis confirmed domain switching from in-plane to out-of-plane polarization for *x* = 0.2. *e*_31,f_ of 19 C/m^2^ was observed for films in the 10% composition range of *x* = 0.2–0.3 using cantilever structures; this *e*_31,f_ was almost the highest value in Pb-free materials and comparable to that of Pb-based ones. These results demonstrate good piezoelectric properties over a compositional range several times broader than the limited MPB range of 1–2%. The innovative concept of reversible domain switching facilitates improved piezoelectric properties over an extended composition range, in a departure from conventional MPB compositions. We believe that the achievement of high environmental sustainability and composition insensitivity in lead-free piezoelectric materials will inspire further exploration of piezoelectric materials, which have been dominated by Pb-based materials near MPB composition for the last 70 years.

## Experimental Section

### Film Preparation

Approximately 2.0 μm thick (1–*x*)(Bi,Na)TiO_3_–*x*BaTiO_3_ films with *x* = 0.06–1.0 were deposited by pulsed laser deposition (PLD) at 675 °C for about 2 h under varying the O_2_ pressure (200 mTorr) using a KrF excimer laser (λ = 248 nm and power of 170 mJ). The targets used for the deposition were prepared via a solid-state reaction of Bi_2_O_3_, Na_2_CO_3_, BaCO_3_, and TiO_2_ powders, with an excess of 20 mol % bismuth oxide and sodium carbonate to compensate for the high volatility of Bi and Na, similar to the process used for sintered ceramics and other film-deposition processes.

(Bi,Na)TiO_3_–BaTiO_3_ films were deposited on (100)-oriented Si single-crystal substrates covered with a Pt electrode, Pt/TiO_2_/SiO_*x*_/(100)Si. To deposit {100}-out-of-plane-oriented textured films, a LaNiO_3_ buffer layer, which can achieve {100}-preferred-oriented textured films independent of the kinds of substrate,^51,52^ was inserted between the (La_0.5_Sr_0.5_)CoO_3_ electrode layer and the (111)Pt/TiO_2_/SiO_*x*_/Si substrates. LaNiO_3_ films were prepared by RF sputtering at 350 °C and subsequent heat treatment at 800 °C, showing the (100)_*c*_ orientation (the subscript *c* indicates pseudocubic cells). (La_0.5_Sr_0.5_)CoO_3_ films were prepared by using PLD to ensure sufficient conductivity of the electrode.

### XRD Analysis

The crystal structures of the prepared films were analyzed using X-ray diffraction (XRD; X’Pert-MRD, Philips, and SmartLab, Rigaku, λ = 0.154 nm). The ω*-*2θ scans were carried out to estimate the lattice parameters by performing 2θ scans while changing the incident angle (ω). The 2θ position of Si (lattice parameter: 5.43) was used as a reference (Coll. Code: 51,688). The film thickness was estimated using wavelength-dispersive X-ray fluorescence (WD-XRF; Axios PW4400/40, PANalytical), and the results were compared to those of a reference sample. The crystal structures of the films under an applied electric field were investigated using a microfocus X-ray diffraction (XRD) setup with a 2D detector (Bruker AXS D8 DISCOVER) by focusing X-rays on the Pt-top electrodes. X-rays were focused onto a Pt-top electrode with ϕ = 200 μm, to which an electric field of 250 kV/cm amplitude was applied, and diffraction patterns were collected by a two-dimensional detector. A collimator with a pinhole with a 100 μm diameter was used.

### Microstructure Analysis

The surface morphology and cross-sectional microstructure were observed by using a field emission scanning electron microscope (FESEM; Hitachi, S-4800) and an atomic force microscope (AFM) (SPA400, SII).

### Electrical Characterization

Pt-top electrodes with ϕ *=* 200 μm were deposited on (Bi,Na)TiO_3_–BaTiO_3_ films via evaporation to measure electric and piezoelectric properties. The ferroelectricity at room temperature for the Pt/(Bi,Na)TiO_3_–BaTiO_3_/(La_0.5_Sr_0.5_)CoO_3_ capacitor was measured by using a ferroelectric tester (TOYO, FCE-1A) at 10 kHz. The electric-field-induced strain was recorded using laser Doppler vibrometers (LDV, Polytec, NLV-2500-5) simultaneously with the *P*–*E* measurements. *e*_31,f_ was determined from the tip displacement of the cantilever using the LDV. The sample length and thickness of the cantilevers are 11.5 mm and 780 μm for the Si substrate and 11.9 mm and 500 μm for the SrTiO_3_ substrate, respectively. The tip displacement was produced by applying a sinusoidal voltage with various amplitudes and a bias of −10 V, which had been polled with a 20 V pulse wave.

## Data Availability

The data supporting the findings of this study are available from the corresponding author upon reasonable request.

## Supporting Information Available

Out-of-plane and in-plane XRD patterns, SEM images, surface morphology, and remanent polarization as a function of measured maximum amplitude (PDF)
