# Supplementary material for: Achieving High Piezoelectric Performance across a Wide Composition Range in Tetragonal (Bi,Na)TiO3–BaTiO3 Films for Micro-electromechanical Systems
Source: ACS Appl Mater Interfaces. 2023 Dec 28;16(1):1308–16. doi: 10.1021/acsami.3c13302 (PMC10788825; doi:10.1021/acsami.3c13302)
Supplement: Supplementary file 1 — am3c13302_si_001.pdf [file am3c13302_si_001.pdf]

Supporting Information

Achieving High Piezoelectric Performance Across a Wide Composition Range in Tetragonal $(Bi,Na)TiO_3$-$BaTiO_3$ Films for Micro Electromechanical Systems

Keisuke Ishihama[1]*, *Takao Shimizu*[1,2], *Kazuki Okamoto*[1], *Akinori Tateyama*[1], *Wakiko Yamaoka*[3], *Risako Tsurumaru*[3], *Shintaro Yoshimura*[3], *Yusuke Sato*[3], *and Hiroshi Funakubo*[1]*

[1] School of Materials and Chemical Technology, Tokyo Institute of Technology, Yokohama 226-8502, Japan

[2] Research Center for Functional Materials, National Institute for Materials Science, Tsukuba 305-0044, Japan

[3] Technical Center, TDK Corporation, Ichikawa 272-8558, Japan

Keywords: piezoelectric films, Pb-free perovskite materials, domain switching

*funakubo.h.aa@m.titech.ac.jp

Contents

Table S1. Lattice parameters obtained from *in-situ* XRD measurement data shown in Figure S6.

Figure S1. (a) Out-of-plane and (b) in-plane XRD profiles of prepared $(Bi,Na)TiO_3$-$BaTiO_3$ (x=0.06-1.0) films on $(100)_c(La_{0.5}Sr_{0.5})CoO_3/(100)_c$ $LaNiO_3$/ Pt/Ti/SiO_x/(100)Si substrates. $LaNiO_3$, $(La_{0.5}Sr_{0.5})CoO_3$, and Pt electrodes were peaks from underlying layer from substrates.

Figure S2. (a) Surface and (b) cross-section SEM images of 0.8BNT-0.2BT film deposited on Si substrate.

Figure S3. Remanent polarization as a function of measurement maximum amplitudes at (a) 1st and (b) 2nd sweep-up data for $(1\text{-}x)(Bi,Na)TiO_3$-$xBaTiO_3$ films with x = 0.06-1.0.

Figure S4. AFM images of (a) x = 0.06, (b) x = 0.2, (c) x = 0.3, (d)x = 0.5, and (e)x = 1.0 of prepared $(Bi,Na)TiO_3$-$BaTiO_3$ films.

Figure S5. Composition, x, dependencies of average roughness for $(1-x)(Bi, Na)TiO_3$-$xBaTiO_3$ films with x = 0.06–1.0. The error bars are the standard deviations.

Figure S6. Peak fitting result of the XRD pattern under applied electric field is shown in Figure 5(b). Three peaks from BNT-BT 200, BNT-BT 002, and LSCO/LNO 200_c were used for fitting.

Table S1. Lattice parameters obtained from *in-situ* XRD measurement data shown in Figure S6.

	**Vc** (-)	***a*** (Å)	***c*** (Å)
w/o	0	3.921	-
w Elec. Field	0.33	3.921	3.949

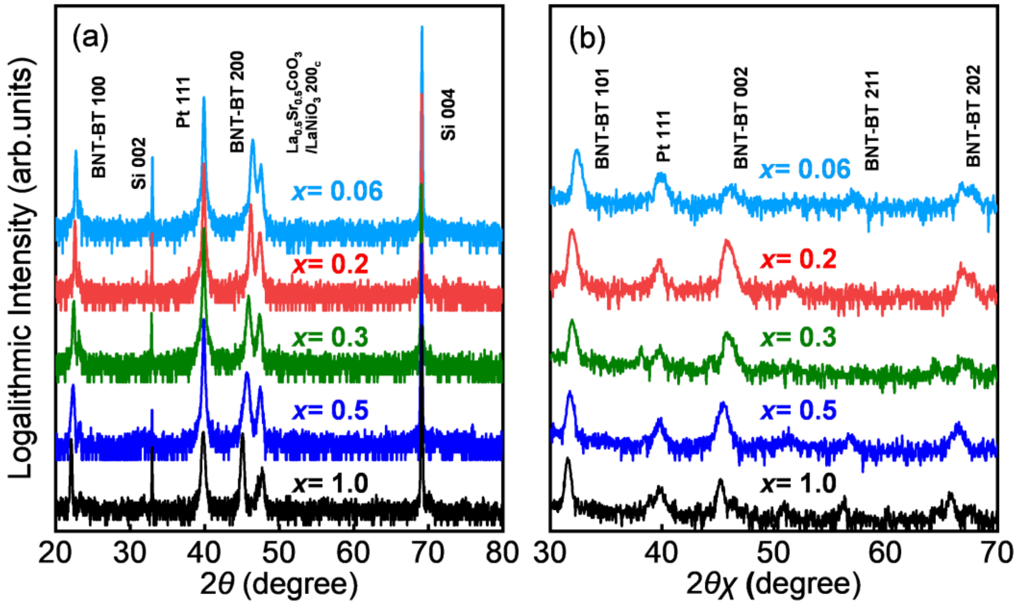

Figure S1. (a) Out-of-plane and (b) in-plane XRD profiles of prepared (Bi,Na)TiO_3-$BaTiO_3$ (x=0.06-1.0) films on $(100)_c$($La_{0.5}Sr_{0.5}$)CoO_3/$(100)_c$ $LaNiO_3$/ Pt/Ti/SiO_x/(100)Si substrates. $LaNiO_3$, ($La_{0.5}Sr_{0.5}$)CoO_3, and Pt electrodes were peaks from the underlying layer from substrates.

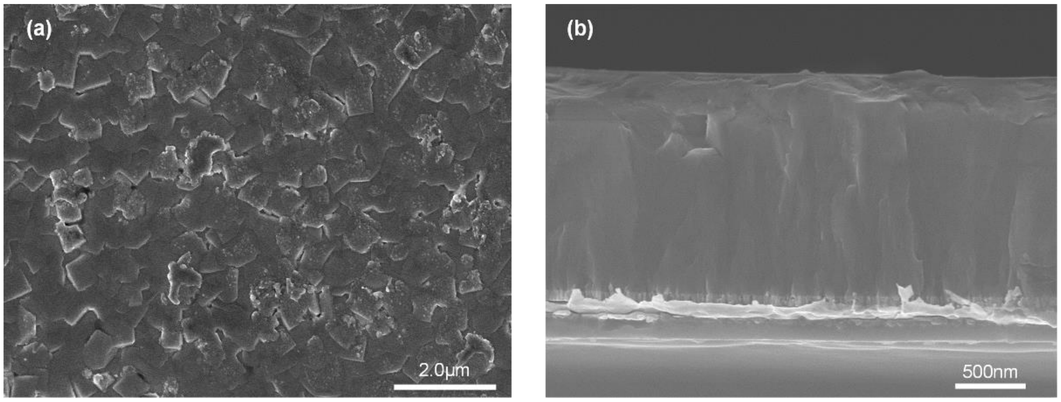

Figure S2. (a) Surface and (b) cross-section SEM images of 0.8BNT-0.2BT film deposited on Si substrate.

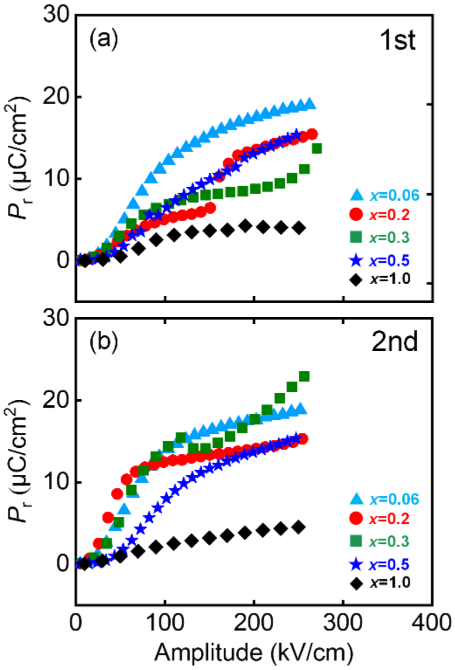

Figure S3. Remanent polarization as a function of measurement maximum amplitudes at (a) 1st and (b) 2nd sweep-up data for (1-x)(Bi,Na)TiO_3-$x$$BaTiO_3$ films with x=0.06-1.0.

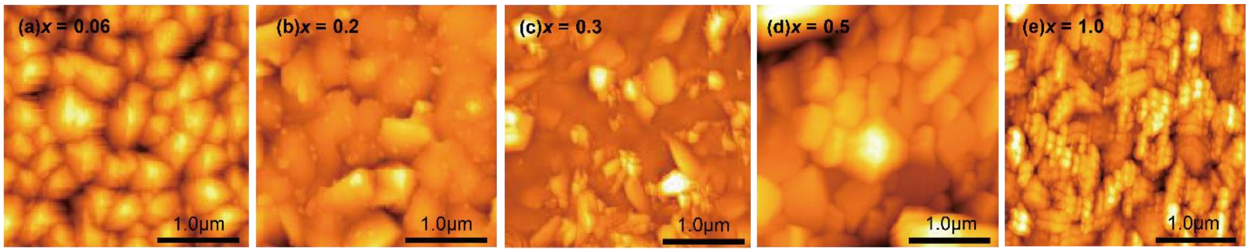

Figure S4. AFM images of (a) $x = 0.06$, (b) $x = 0.2$, (c) $x = 0.3$, (d)$x = 0.5$, and (e)$x = 1.0$ of prepared (Bi,Na)TiO_3-$BaTiO_3$ films.

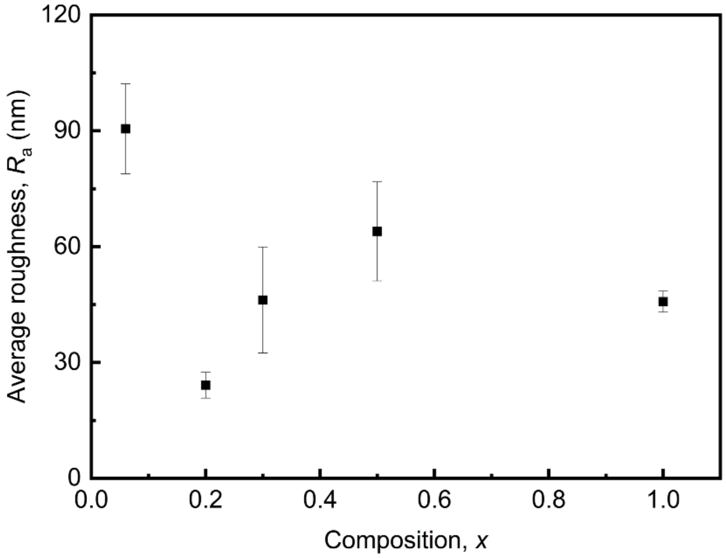

Figure S5. Composition, x, dependencies of average roughness for $(1-x)$(Bi, Na)TiO_3-$x$$BaTiO_3$ films with $x = 0.06$–1.0. The error bars are the standard deviations.

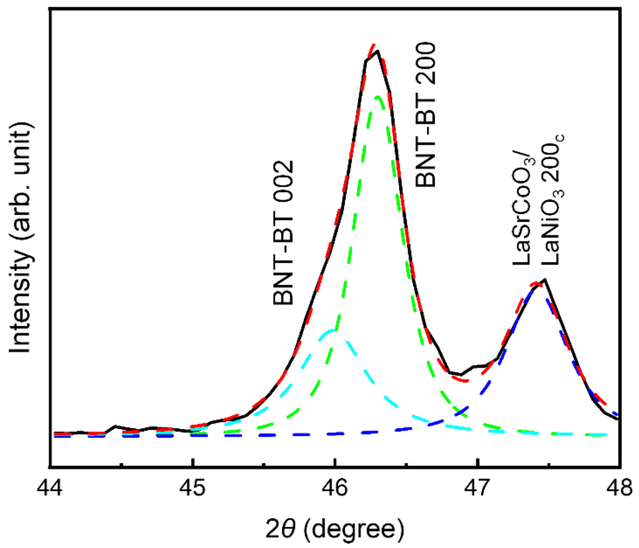

Figure S6. Peak fitting result of XRD pattern under applied electric field illustrated in Figure 5(b). Three peaks from BNT-BT 200, BNT-BT 002, and LSCO/LNO 200_c were used for the fitting.
